# Novel epitope-based diagnostic probes selected by *phage display* for the serological detection of HDV

**DOI:** 10.1590/0074-02760250343

**Published:** 2026-06-12

**Authors:** Mariana Araújo Costa, Rayany Cristina de Souza, Tárcio Peixoto Roca, Adrhyan Araújo, Luiz Fellype Alves de Souza, Rutilene Barbosa Souza, Rafaela Sabatini, Bruno Silva Andrade, Iara Pereira Soares, Douglas Carvalho Caixeta, Marco Guevara-Vega, Ildercílio Mota de Souza Lima, Ana Maísa Passos-Silva, Mario Machado Martins, Abel Dib Rayashi, Daniel Archimedes da Matta, Deusilene Vieira, Luiz Ricardo Goulart, Robinson Sabino-Silva, Fabiana de Almeida Araújo Santos

**Affiliations:** 1 Universidade Federal de Uberlândia, Instituto de Biotecnologia, Laboratório de Nanobiotecnologia Prof Dr Luiz Ricardo Goulart Filho, Uberlândia, MG, Brasil; 2 Universidade Federal de Uberlândia, Centro de Inovação em Diagnóstico Salivar e Nanobiotecnologia, Instituto de Ciências Biomédicas, Departamento de Fisiologia, Uberlândia, MG, Brasil; 3 Secretaria de Saúde do Estado do Acre, Laboratório Central de Saúde Pública do Acre, Rio Branco, AC, Brasil; 4 Universidade Federal do Acre, Laboratório de Biologia Celular e Molecular Aplicada, Rio Branco, AC, Brasil; 5 Fundação Oswaldo Cruz-Fiocruz, Laboratório de Hepatites Virais, Rio de Janeiro, RJ, Brasil; 6 Fundação Oswaldo Cruz-Fiocruz, Laboratório de Virologia Molecular, Porto Velho, RO, Brasil; 7 Fundação Hospital Estadual do Acre, Centro de Infectologia Charles Merieux & Laboratório Rodolphe Merieux, Rio Branco, AC, Brasil; 8 Universidade Federal da Bahia, Programa de Pós-Graduação em Medicina e Saúde, Salvador, BA, Brasil; 9 Universidade Estadual do Sudoeste da Bahia, Departamento de Ciências Biológicas, Laboratório de Bioinformática e Química Computacional, Jequié, BA, Brasil

**Keywords:** hepatitis delta virus, phage display, diagnostic, ELISA

## Abstract

**BACKGROUND:**

Hepatitis delta virus (HDV) is associated with the worst prognosis among viral hepatitis infections; however, it remains largely underdiagnosed, particularly in endemic developing regions, underscoring the need for new, accessible serological methods for large-scale screening.

**OBJECTIVES:**

To develop HDV-mimetic molecules using *phage display* for application in immunodiagnostic platforms.

**METHODS:**

HDV-mimetic peptides were selected via *phage display* biopanning, sequenced and screened by phage-enzyme-linked immunosorbent assay (ELISA). Based on the sequences of these peptides, a recombinant protein (rHDV) was constructed and employed in an ELISA. The test was validated using 87 anti-HDV-positive samples and 93 hepatitis B virus (HBV) control samples collected from a public diagnostic laboratory in the Amazon region. Statistical analyses were performed to evaluate the diagnostic performance of the synthetic peptides and the recombinant protein.

**FINDINGS:**

Overall, rHDV exhibits a sensitivity of 74.71%, specificity of 97.85%, and area under the curve (AUC) of 0.8906. In HDV ribonucleic acid (HDV RNA)-positive patients, diagnostic performance improved, with a sensitivity of 88.0%, specificity of 98.92%, and AUC of 0.96.

**MAIN CONCLUSIONS:**

These findings highlight that the rHDV protein used in ELISA effectively discriminated HDV-infected individuals from patients monoinfected with hepatitis B. This demonstrates the potential of rHDV as an effective, rapid, and low-cost tool for HDV detection for broader HDV screening.

The hepatitis delta virus (HDV) is a small, spherical virus with a negative-sense, single-stranded circular RNA genome that encodes two forms of the hepatitis delta antigen (HDAg): the small (S-HDAg) and large (L-HDAg) proteins. A distinctive feature of HDV is its viral envelope, which consists of the hepatitis B virus (HBV) surface antigen (HBsAg).^1^ HDV is often classified as a satellite virus of HBV, as it cannot efficiently complete its replication cycle in humans, without HBV.^2^

HDV is highly diverse, comprising eight genotypes with intergenotypic homology ranging from 80% to 85% and distinct geographic distributions. Among these, HDV-1 is the most prevalent globally and may be the precursor of all other genotypes. In contrast, HDV-3, which is exclusive to South America, shows the greatest divergence.^3^ ^,4^ Despite its global significance, the prevalence of HDV remains uncertain, with estimates of infected individuals ranging from 12 to 72 million.^5^

Chronic hepatitis D infection is the most severe form of viral hepatitis, with a high risk of progression to liver cirrhosis, hepatocellular carcinoma and liver-related death.^5^ ^,6^ Current guidelines recommend HDV testing for all patients with chronic HBV infection.^7^ However, there is a considerable lack of testing, particularly in developing countries where the virus is endemic,^8^ leading to its underestimation.^7^ Notably, high HDV prevalence has been reported in the Brazilian Amazon region,^9^ ^,10^ ^,11^ ^,12^ ^,13^ with 72.4% of all the cases reported between 2000 and 2024 occurring in the Northern region of Brazil, which is also the source of the samples in this study.^14^ These challenges highlight the urgent need for affordable and accessible tools for HDV screening.

*Phage display* technology offers a promising approach to diagnostic platforms. This method utilises a combinatorial library of bacteriophages displaying diverse peptides that bind specific targets with high affinity and specificity.^15^ ^,16^ The selection process, known as biopanning, enriches high-affinity molecules by eliminating those with low binding potential.^17^ ^,18^ These high-affinity bacteriophages can be amplified and sequenced to identify the peptide sequences they encode^17^ and further applied to diagnostic platforms. In this context, we used *phage display* technology to select and synthesise HDV mimetic molecules recognised by anti-HDV antibodies. These molecules were integrated into an immunoenzymatic platform designed for the detection of hepatitis delta.

## MATERIALS AND METHODS

*Ethical approval statement* - All procedures adhered to established ethical guidelines and regulations. The study was approved by the Ethics Committee of the Federal University of Uberlândia, which granted a waiver for the Informed Consent Form due to the residual nature of the samples and the absence of identifiable patient information (CAAE: #44208621.2.0000.5152).

*Samples* - All procedures adhered to established ethical guidelines and regulations. A total of 200 blood serum samples were obtained from a public diagnostic laboratory in the Amazon region at the Central Laboratory of Public Health of Acre (LACEN-AC). The samples were classified and coded by LACEN-AC as part of routine testing, using the enzyme-linked immunosorbent assay (ELISA) kit ETI-AB-DELTAK-2 (Diasorin, Saluggia, Italy) for anti-HDV detection. The samples were then grouped based on their serological status for viral hepatitis:

Group 1: negative for HBsAg, total anti-HBc, anti-HBs, and anti-HCV (10 samples).

Group 2: negative for HBsAg, anti-HCV, and total anti-HBc, but positive for anti-HBs (10 samples).

Group 3 (HBV Control): positive for HBsAg and negative for anti-HDV (93 samples).

Group 4 (anti-HDV-positive): positive for both HBsAg and anti-HDV (87 samples).

*Coupling of IgGs in protein G magnetic beads* - To begin the *phage display* selection cycles, we employed magnetic protein G-coupled Dynabeads™ (Life Technologies Corporation®, Carlsbad, CA, USA) for IgG antibody coupling. The protocol was performed following the manufacturer's guidelines, with some modifications. First, 2 × 10^9^ bead particles (100 µL of the stock) were washed three times with phosphate-buffered saline (PBS) to activate the microspheres. Then, 100 µL of pooled sample (10 µL from 10 patients) from each participant group (Group 1, Group 2, Group 3, and Group 4) was added to four different tubes and incubated under agitation for 40 minutes at room temperature. The antibody-adsorbed beads were separated using a magnet stand (DynaMag™, Washington Mills North Grafton, Inc., North Grafton, MA, USA) to remove the supernatant (non-binding antibodies). The attached beads were subsequently washed three times in PBS supplemented with 0.05% Tween 20 (0.05% PBS-T) to remove any non-binding antibodies.

**Fig. 1:**
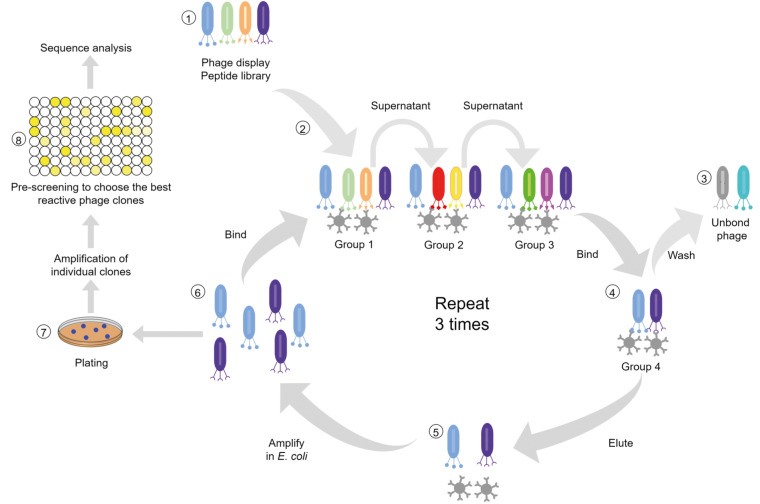
a scheme of biopanning. (1) The phage library was incubated with Dynabeads™ Protein G and, sequentially, (2) with immunoglobulin G (IgG) antibodies for three negative selections. (3) Unbound phages were washed, and (4) one positive selection was performed. (5) Phages recognised by the anti-hepatitis delta virus (HDV) antibody (IgG) Dynabeads™ were eluted, (6) and amplified from *Escherichia coli* ER2738 for three rounds. (7) The eluted phages were plated, collected, and amplified, and (8) the best reactive phage clones were selected by enzyme-linked immunosorbent assay (ELISA) and sequence analysis.

*Biopanning* - The biopanning process involved selecting and amplifying HDV mimetic peptides. For selection, 10 µL of viral suspension (2 × 10^11^ phages) from a Ph.D.-C7C library of phage-fused peptides (New England Biolabs, Beverly, MA, USA) was diluted in 190 µL of 0.1% PBS-T to select ligands for IgG from HDV-positive patients. The phage library contains seven random amino acids expressed in the pIII region of the bacteriophage, flanked by a pair of cysteine residues that form a disulfide bond during phage assembly. Selection was performed using 20 µL of protein G microspheres bound to immunoglobulins from the four different groups. Three negative selections were performed for this purpose. The phage library was successively incubated (for 30 min at room temperature) with pooled samples from Group 1, and the unbound phage particles were then transferred to pooled samples from Groups 2 and 3, followed by positive selection with pooled samples from the anti-HDV-positive Group 4. The beads were washed 10 times with 0.1% PBS-T, after which the supernatant was discarded. Acid elution was performed by adding 500 µL of 0.2 M glycine (pH 2.2) and incubating the solution for 10 min. The supernatant was removed and neutralised with 75 µL of Tris-HCl (pH 9.1). After the selection phase, the elutes were amplified using the *Escherichia coli* ER2738 strain (New England Biolabs, Beverly, MA, USA), which was purified with 20% polyethylene glycol (PEG)/2.5 M NaCl and suspended in PBS. Three rounds of biopanning were performed. After each round, titration was performed to determine the input and output of viral particles during the selection cycle. For all titrations, 1 µL of phage was diluted in 9 µL of Luria-Bertani (LB) culture medium. Non-amplified eluates were diluted from 10^-1^ to 10^-4^, and amplified phages were diluted from 10^-8^ to 10^-11^. The diluted solutions were incubated with 200 µL of *E. coli* ER2738 strain in mid-log phase (OD600 ~0.5) for 5 min and plated in LB medium supplemented with 840 mM isopropyl β-D-1-thiogalactopyranoside (IPTG) and 49 mM 5-bromo-4-chloro-3-indolyl β-D-galactopyranoside (X-gal), combined with 3 mL of Top Agar. After incubating for 18 hours at 37ºC, the phage-infected *E. coli* ER2738 colonies appeared blue^16^ (Fig. 1).

*DNA extraction and sequencing of phages* - The phages were isolated by third-round titration and grown overnight in deep-well plates supplemented with 1.2 mL of early-log-phase *E. coli* ER2738 culture (OD600 ~0.03). DNA extraction was performed as previously described.^16^ ^,19^ DNA sequencing was carried out using the DNA from the selected phages with a BigDye® Terminator v3.1 Cycle Sequencing Kit on an ABI 3730xl Genetic Analyzer (Applied Biosystems, Foster City, California) at BPI Biotecnologia® (Botucatu, São Paulo, Brazil). The peptide sequences were deduced from the nucleotide sequences and analysed using ExPASy (https://web.expasy.org/translate/).

*Phage-ELISA* - An ELISA was performed to analyse the reactivity of the clones with IgGs from anti-HDV-positive patients against the hepatitis B control. Microtiter plates (NUNC Maxisorp, Thermo Fisher Scientific, Waltham, MA, USA) were coated with a 1:500 dilution of anti-M13 monoclonal antibody (GE Healthcare, Chicago, IL, USA) in 50 mM bicarbonate buffer (pH 9.6) for 16 h at 4ºC. The plates were then washed with PBS and blocked with 3% bovine serum albumin (BSA) in PBS-T for 1 h at 37ºC. Afterward, the plates were washed three times with 0.05% PBS-T and incubated with phage supernatant for 1 h at 37ºC. The plates were subsequently washed six times and incubated with a pool of serum from hepatitis B control and anti-HDV-positive patients, diluted 1:100 in 0.05% PBS-T, for 1 h at 37ºC. Following six additional washes, the plates were incubated with anti-human IgG conjugated with horseradish peroxidase (HRP) at a 1:5000 dilution. After six washes, antigen/antibody binding was detected by adding ortho-phenylenediamine (OPD) buffer at 1 mg/mL with 3% H_2_O_2_ (Sigma Chemical Co., St. Louis, MO, USA). The reaction was stopped by adding 4 N sulfuric acid. Reactivity was measured using a plate reader at 492 nm. The most promising phages were subjected to another round of phage ELISA with serum from individual patients (10 positives and 10 negatives for hepatitis D). We also assessed two blocking agents (BSA or skim milk), three serum dilutions (1:100, 1:250, and 1:500), and two different phage concentrations (10^10^ and 10^11^ pfu/well).

*Peptide design and synthesis* - The sequenced phage clones were aligned^20^ to the L-HDAg sequence, a protein of HDV obtained from GenBank (AVO03797.1), using Clustal Omega for the design of synthetic peptides. Additionally, B cell epitope antigenicity from the L-HDAg sequence was analysed using linear epitope prediction (version 2.0)^21^ ^,22^ to identify potential epitope regions. The epitope regions that aligned with the most reactive clones were selected for peptide sequence design. Upon alignment of the clone sequences with the L-HDAg protein, several clones aligned within the same region. Consequently, the native sequence of the protein was used to design three synthetic peptides through GenScript, USA, Inc. (Piscataway, NJ, USA). The selected peptides were HDAg1 (ACKDGEGAPCGGGSAETVES), HDAg2 (ACMEVDSGPCGGGSAETVES), and HDAg3 (KDGEGAPPAKRARTDQMEVDSGP). HDAg1 and HDAg2 were constructed with 20 residues, contained a disulfide bridge (between residues 2-10), were acylated at the N-terminal, and were amidated at the C-terminal. HDAg3 was constructed with 23 residues, was acylated at the N-terminal, was amidated at the C-terminal, and did not contain a disulfide bridge. The physicochemical properties of the peptides were calculated using ExPASy (https://web.expasy.org/protparam/). Furthermore, an analysis of the conservation of the HDAg3 peptide was performed by aligning it with different L-HDAg sequences from distinct genotypes deposited in the GenBank database of the National Centre for Biotechnology Information (NCBI). The alignment was conducted using the ClustalW algorithm in the Molecular Evolutionary Genetics Analysis (MEGA, version 11) software.

*3D modelling* - The L-HDAg sequence (AVO03797.1) was submitted to the artificial intelligence (AI) generated tool trRosetta server (http://yanglab.nankai.edu.cn/trRosetta/) for 3D modelling.^23^ Additionally, the same sequence was submitted to the protein homology modelling server Phyre2 (http://www.sbg.bio.ic.ac.uk/phyre2/html/page.cgi?id=index), which used the 1A92 crystal structure from the oligomerisation domain of L-HDAg as a template.^24^ Both structures, generated by AI and homology tools, were structurally aligned to identify conserved domains using PyMOL 2.5 (Schrödinger, LLC). Subsequently, the HDAg1 and HDAg2 sequences were submitted for three-dimensional homology modelling against the 3D model of L-HDAg (trRosetta) using The Pepitope Server (http://pepitope.tau.ac.il/).

*Recombinant protein design, expression and purification* - The alignment of phage clones also revealed that sequences up to the 118-aa portion of L-HDAg aligned with specific phage clones, particularly HD28 (DTTLSDN) and HD16 (DTTLHLG). These clones indicated the presence of additional immunogenic sequences beyond aa 85, which could potentially improve antibody recognition. Based on these findings, we decided to expand the target region to span aa 63-118 and design a recombinant protein (rHDV) that utilises the larger region.

rHDV was synthesised using the pET-22b(+) cloning vector by GenOne Biotech (Rio de Janeiro, Brazil). This vector was constructed from the L-HDAg viral protein sequence (residues 63-118) with a molecular weight of 12.4 kDa. The plasmid was subsequently transformed into *E. coli* BL21 (DE3) pLysS cells, which were incubated on an LB agar plate supplemented with 100 μg/mL ampicillin overnight at 37ºC. A single colony from this plate was inoculated into a 10 mL LB culture (100 μg/mL ampicillin), which was grown for 12 h at 37ºC and 250 rpm. The overnight culture was then used to inoculate 600 mL of LB (100 μg/mL ampicillin), and the mixture was incubated (OD600 = 0.8). After growth, 1 mM IPTG was added, and the mixture was incubated for 5 h at 37ºC and 250 rpm. The cells were collected by centrifugation (3,500 × g for 15 min, 4ºC) and resuspended in PBS supplemented with 0.25% lysozyme. The cells were lysed by thermal shock and sonication for 30 s, repeated three times under 0.55 cycles at 55% amplitude. After sonication, the cells were centrifuged at 10,000 × g at 4ºC for 30 min. The supernatant was discarded, and urea buffer (8 M urea and 25 mM Tris-HCl, pH 8.0) was added to the precipitated cells. The mixture was centrifuged again at 10,000 × g and 4ºC for 20 min, after which the precipitate was discarded. The supernatant was purified via HPLC (Shimadzu, Kyoto, Japan) and quantified using sodium dodecyl sulphate-polyacrylamide gel electrophoresis (SDS-PAGE) with ImageJ software, with BSA (1 mg/mL) as a reference.

*ELISA with synthetic peptides and recombinant protein* - The first ELISA with synthetic peptides (HDAg1, HDAg2, and HDAg3) was performed to determine the optimal conditions for serum dilution (1:100 and 1:250) and peptide concentration (200 ng/well and 1 μg/well). For this purpose, an ELISA plate (Greiner, Frickenhausen, Germany) was coated in duplicate with HDAg1, HDAg2, or HDAg3, diluted in carbonate buffer (0.1 M NaHCO_3_, pH 8.6) overnight at 4ºC. The plate was then washed with 0.05% PBS-T and blocked with 0.05% PBS-T containing 5% skim milk for 1 h. The plate was washed three times and incubated for 1 h at 37ºC with individual sera (diluted 1:100 and 1:250 in 0.05% PBS-T). Afterward, the wells were washed six times and incubated with anti-human IgG HRP (GE Healthcare, Chicago, IL, USA) diluted 1:5000 in 0.05% PBS-T for 1 hour at 37ºC. The plate was washed six times and developed with OPD, and the absorbance was measured at 492 nm. Another ELISA protocol was used to compare the reactivity of HDAg3 and rHDV in anti-HDV-positive patients (87 samples) and HBV controls (93 samples). A plate was coated in duplicate with HDAg3 (200 ng/well) and rHDV (0.05 μg/well), diluted in carbonate buffer (0.1 M NaHCO_3_, pH 8.6) overnight at 4ºC. The wells were then washed with 0.1% PBS-T and blocked with 0.1% PBS-T containing 5% skim milk for 1 h. The plate was washed three times and incubated for 1 h at 37ºC with individual sera (diluted 1:250 in 0.1% PBS-T). The wells were washed six times, followed by incubation with anti-human IgG HRP and anti-human IgM (μ-chain specific) HRP (Sigma-Aldrich, St. Louis, MO, USA) diluted 1:5000 in 0.1% PBS-T for 1 h at 37ºC. The plate was washed six times and developed with OPD, and subsequent readings were taken at 492 nm.

*HDV RNA detection and sequencing* - Samples from Group 4 (anti-HDV-positive) that had sufficient volume were selected for HDV RNA detection. Total nucleic acids were extracted from 100 μL of serum using the Loccus Extracta 32 equipment (Loccus, Brazil), following the manufacturer's instructions. HDV RNA was detected via reverse transcription quantitative polymerase chain reaction (RT-qPCR) in 50 samples, as described previously.^25^ The cycle thresholds (Cts) were analysed, and samples with viral target Cts ≤ 30 were selected for the sequencing protocol. The sequencing protocol involved cDNA synthesis using the SuperScript III Two-Step kit (Invitrogen). To characterise the genotypes of HDV, PCR was performed using the primers 853IU (5′ CGGATGCCCAGGTCGGACC 3′) and 1302OD (5′ GGATTCACCGACAAGGAGAG 3′), which have been previously described^26^ and are known to amplify a 450-bp fragment of the HDV genome. Nested PCR was then performed using primers HDV-E (5′ GAGATGCCATGCCGACCCGAAGAG 3′) and HDV-A (5′ GAAGGAAGGCCCTCGAGAACAAGA 3′), also previously described.^27^ The PCR products were analysed by gel electrophoresis, followed by purification with ExoSAP (Cellco, New York, NY, USA). Sequencing was carried out by the Rede de Plataformas Tecnológicas FIOCRUZ RPT09F-FIOCRUZ/RO using the automated Sanger sequencer SeqStudio (Applied Biosystems, Waltham, MA, USA). The sequences were analysed using BLAST (https://blast.ncbi.nlm.nih.gov/Blast.cgi) for genotype confirmation.

*Statistical analysis* - The reactivity ratio was calculated by comparing the absorbances of the positive-HDV samples to the control samples. To determine differences in peptide and recombinant protein reactivity among the groups, the data were considered non-normally distributed, and an unpaired t-test with a Mann-Whitney test was performed. Additionally, sensitivity, specificity, cutoff values, and the area under the curve (AUC) were calculated based on receiver operating characteristic (ROC) curve analysis. All analyses were performed using GraphPad Prism 7 software (GraphPad Software, Inc., San Diego, CA). Values of p < 0.05 were considered statistically significant, and the results are expressed as the mean ± standard deviation (SD).

## RESULTS

**Fig. 2:**
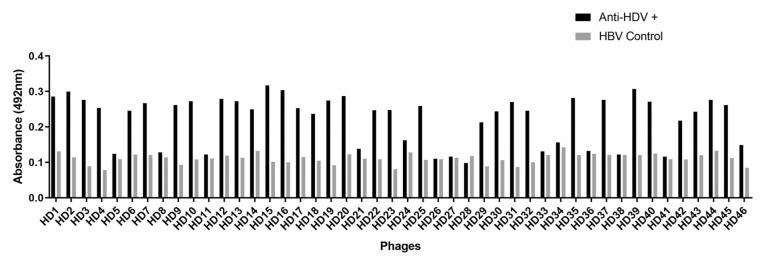
phage-enzyme-linked immunosorbent assay (ELISA) results for 46 selected phage clones from biopanning with anti-hepatitis delta virus (Anti-HDV+) and hepatitis B virus (HBV) control serum pools.

*Sequence analysis and Phage-ELISA* - After three rounds of biopanning, 80 samples presented valid sequences (without sequence errors), with 46 unique sequences [Supplementary data (Table I)]. These 46 phages were subjected to an immunoreactivity test using phage-ELISA (Fig. 2). Some clones exhibited substantial reactivity, with ratios ranging from 2-3 in 25 clones when absorbances from pooled samples of anti-HDV-positive patients were compared to those from HBV control patients. Additionally, six clones demonstrated a reactivity ratio of ≥ 3.

**Fig. 3:**
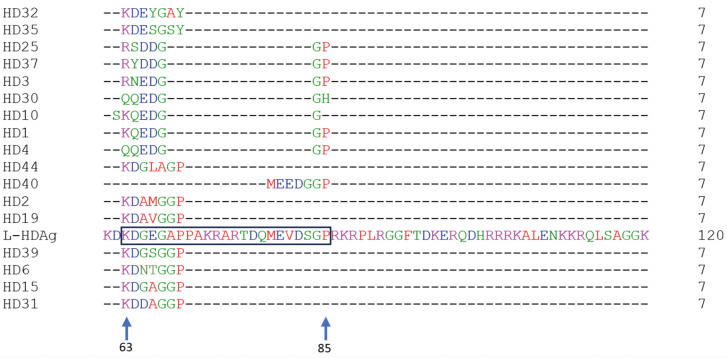
a portion of the amino acid sequence of the phage clones was aligned with the large hepatitis delta antigen (L-HDAg) protein sequence (AVO03797.1). The selected region (black box) was used to design the peptides HDAg1, HDAg2, and HDAg3.

*Alignments of phage clones with the reference HDV protein sequence* - The alignment of phage clone sequences with the L-HDAg protein revealed that several clones, including HD32, HD35, HD25, HD37, HD3, HD30, HD10, HD1, HD4, HD44, HD40, HD2, HD19, HD39, HD6, HD15, and HD31, aligned with the 63-85 region of the L-HDAg protein (Fig. 3). This region was identified as an immunogenic epitope through B-cell epitope prediction analysis [Supplementary data (Fig. 1)], suggesting its potential for diagnostic applications.

To further enhance diagnostic performance, we expanded the target region to span amino acids 63-118. The alignment of the L-HDAg protein with phage clone sequences revealed alignment up to the 118-aa portion with specific clones, particularly HD28 (DTTLSDN) and HD16 (DTTLHLG). These findings indicated that extending the region beyond aa 85 would include additional immunogenic sequences, potentially improving antibody recognition. Using this expanded region, we designed a recombinant protein (rHDV).

**Fig. 4:**
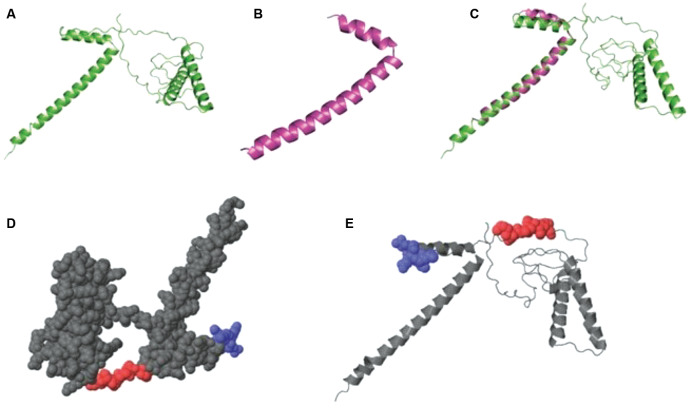
3D structure of large hepatitis delta antigen (L-HDAg): (A) modeled by trRosetta; (B) modeled by Phyre2; (C) structural comparison between L-HDAg models from Phyre2 (pink) and trRosetta (green). The 3D-aligned region spans from the amino acid GLY12 to LYS61. Alignments in 3D structures using the sequence of L-HDAg (gray) were performed by PepSurf. Peptides were mapped onto L-HDAg; HDAg1 is represented in blue, and HDAg2 is represented in red. (D) Space-filling model. (E) Ribbon model.

*In silico analyses: L-HDAg modelling and alignment* - The trRosetta model of the L-HDAg structure included the entire sequence, revealing a fully compact and helical conformation. Structural alignment between the AI-generated model and homologous L-HDAg models demonstrated that the region of the L-HDAg sequence spanning amino acids GLY12 to LYS61, as modelled in Phyre2, closely resembles the trRosetta structure. This similarity suggests that this region may represent a more conserved portion of the structure. Additionally, alignment of the 3D structure of L-HDAg with the peptides HDAg1 and HDAg2 indicated that the selected epitopes effectively mimic the immunogenic region of L-HDAg (Fig. 4). Furthermore, we evaluated the conservation of the designed HDAg3 peptide across HDV sequences [Supplementary data (Figs 2-4)]. The analysis revealed that the HDAg3 peptide exhibited similarity to all regions of the L-HDAg protein investigated, reinforcing its potential as a conserved epitope across diverse HDV sequences.

**Fig. 5:**
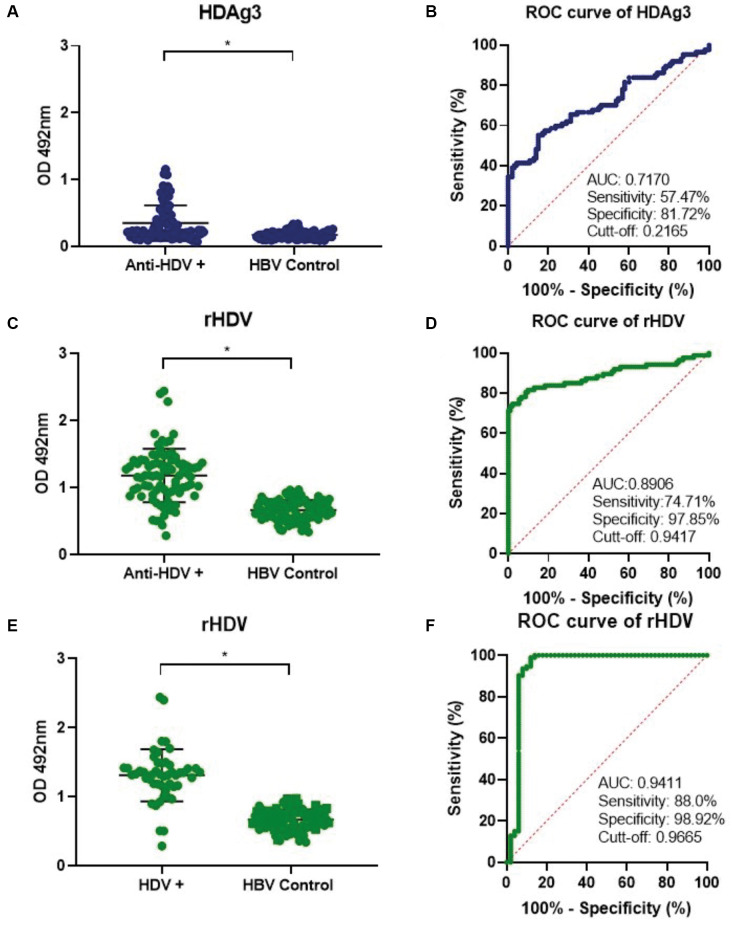
analysis of human serum reactivity using HDAg3 and rHDV with enzyme-linked immunosorbent assay (ELISA), including sensitivity, specificity, and under the curve (AUC) analysis to compare hepatitis delta (HDV) and hepatitis B virus (HBV) control samples. (A) Reactivity of the synthetic peptide HDAg3; (B) Receiver operating characteristic (ROC) curve analysis for HDAg3; (C) Reactivity of the recombinant protein rHDV; (D) ROC curve analysis for rHDV. (E) Reactivity of the recombinant protein rHDV with HDV ribonucleic acid (HDV-RNA)-positive patients and HBV control samples. (F) ROC curve analysis. Note: (*) Statistically significant difference.

*Immunoreactivity of the synthetic peptides and recombinant protein in serum* - The reactivity of the three synthetic peptides (HDAg1, HDAg2, and HDAg3) was compared via ELISA, and we observed that the HDAg3 peptide exhibited superior reactivity. Therefore, the HDAg3 peptide was selected for use in subsequent experiments. Two different serum dilutions (1:100 and 1:250) and two HDAg3 concentrations (200 ng/well and 1 μg/well) were evaluated using 10 anti-HDV-positive serum samples and 10 HBV control samples for test standardisation [Supplementary data (Fig. 5)]. Both serum dilutions and antigen concentrations showed comparable and satisfactory performance in differentiating anti-HDV positive from HBV controls. Given the equivalent performance, the lower antigen concentration (200 ng/well) and the higher serum dilution (1:250) were selected to minimise reagent and human serum consumption. As a result, the synthetic peptide HDAg3 successfully differentiated pooled serum samples from anti-HDV-positive patients and hepatitis B patients.

In this context, we selected HDAg3 for additional analysis using individual sera from 87 positive anti-HDV samples and 93 HBV control samples; the results showed 57.47% sensitivity, 81.72% specificity, and an AUC of 0.717 (Fig. 5A-B). Subsequently, we assessed reactivity using an ELISA test based on the recombinant protein. The results demonstrated an increase in detection parameters, with a sensitivity of 74.71%, specificity of 97.85%, and an AUC of 0.8906 when utilising the same serum samples (Fig. 5C-D). This result highlights the benefit of using the recombinant protein rHDV.

A total of 50 serum samples from patients with confirmed active HDV infection (HDV RNA positive) and 93 HBV control samples were evaluated using the rHDV recombinant protein in an ELISA format. This evaluation yielded a sensitivity of 88.0% and a specificity of 98.92%, with an AUC of 0.9665 (Fig. 5E-F). Additionally, genotype analysis of these samples classified 28 patients as genotype 3 (HDV-3), as detailed in Supplementary data (Table II).

## DISCUSSION

A novel immunodiagnostic platform for hepatitis D detection was developed based on HDV antigenic epitopes selected through *phage display*. This platform is designed to detect anti-HDV antibodies and has the potential to enhance hepatitis D detection, particularly in resource-limited areas, by enabling early diagnosis, facilitating timely treatment initiation, and consequently contributing to improved patient outcomes. The recombinant protein, rHDV, was evaluated in an active infection scenario using 50 serum samples from patients with active hepatitis delta and 93 serum samples from hepatitis B patients. The rHDV demonstrated a high AUC of 0.9665, with 88% sensitivity and 98.92% specificity. When tested with 87 serum samples from anti-HDV-positive hepatitis delta patients and the same 93 serum samples from hepatitis B patients, rHDV, applied in an ELISA-based immunodiagnostic platform, achieved an AUC of 0.8906, with 74.71% sensitivity and 97.85% specificity.

HDV infection is a neglected disease with a high prevalence in low- and middle-income countries (LMICs).^28^ In Brazil, hepatitis D is prevalent in the Amazon region, particularly affecting isolated riverside and indigenous populations.^9^ Due to the limited availability of diagnostic tests for hepatitis D in LMICs, disease incidence is often underestimated, and timely diagnosis is hindered, leading to delays in treatment initiation.^8^ ^,29^ This study employed *phage display* technology to establish a screening platform based on HDV mimetic molecules that interact with anti-HDV antibodies for application in HDV immunodiagnostic platforms. Phage reactivity was screened in the phage-ELISA to select the best-performing clones for differentiating hepatitis delta (anti-HDV-positive) patients from hepatitis B patients. Phage-ELISA was also used to develop a novel diagnostic platform for other diseases in human serum.^30^ We performed phage-ELISA with pooled samples and validated the novel HDAg3 synthetic peptide and recombinant protein based on selected phage sequences.

The sequences of the peptide phages were aligned with L-HDAg using the Clustal Omega alignment tool. The L-HDAg protein has previously been used as a diagnostic target in other studies. A lateral flow test for detecting anti-HDV antibodies utilised recombinant L-HDAg with a 25 kDa His-tagged protein in serum and plasma.^31^ Another serological assay for detecting IgG against HDV, performed with the ARCHITECT immunoassay instrument, was tested on 15 RNA HDV-positive samples using a peptide from the overlapping region of L-HDAg (aa 64-85), detecting 14 out of the 15 samples.^32^

L-HDAg is an immunogenic protein that induces oxidative stress and activates NF-κB and STAT-3, leading to the dysregulation of inflammation, apoptosis, and immune system invasion.^33^ The selected sequence of L-HDAg used in the present study was an epitope region,^22^ identified by Bepipred-Linear Epitope Prediction 2.0. The 3D structure of L-HDAg was not available in the RCSB Protein Data Bank. This study also pioneered the modelling of the L-HDAg protein using the TrRosetta and Phyre2 programs. As depicted in Supplementary data (Fig. 1), the synthetic peptides overlapped in the epitope region of L-HDAg according to Linear Epitope Prediction 2.0 analysis. This amino acid region (63-85) of the L-HDAg protein was previously validated as an immunogenic region,^32^ ^,34^ ^,35^ which confirms the potential of rHDV as a molecule for use in platforms to diagnose hepatitis delta.

The reactivity of the peptides HDAg1, HDAg2, and HDAg3 was evaluated via ELISA, with HDAg3 effectively detecting hepatitis delta disease in serum samples. In this large-scale study, the HDAg3-based platform was validated using samples from 87 hepatitis delta patients (anti-HDV-positive) and 93 hepatitis B patients as controls, achieving a sensitivity of 57.47%, specificity of 81.72%, and an AUC of 0.7170. The HDAg3 sequence was further modified to construct the recombinant protein rHDV. Subsequently, an ELISA using the same samples demonstrated a sensitivity of 74.71%, specificity of 97.85%, and an AUC of 0.8906. These results suggest that the recombinant protein has significant potential for use in clinical screening tests to help control the spread of the disease.

Commercial ELISA kits for HDV detection are available, but they are expensive and have limited market availability, mainly in LMICs.^8^ ^,36^ In Brazil, only one registered brand currently provides this technology.^37^ In contrast, our in-house rHDV-based ELISA offers a cost-effective alternative, making it a more affordable option compared to the commercial kit. Although its sensitivity and specificity are lower than the commercial kit's 98% for both metrics, the in-house assay serves as a viable screening tool, particularly in resource-limited regions, where HDV incidence is high, and access to commercial kits is constrained.

Anti-HDV antibodies are reliable markers of virus exposure in immunocompetent individuals,^5^ while the detection of HDV RNA through molecular techniques remains the gold standard for confirming active infection. In our study, the rHDV protein-based ELISA demonstrated promising performance, achieving a sensitivity of 88.0%, a specificity of 98.92%, and an AUC of 0.9665 when tested with HDV RNA-positive samples. These results highlight the assay's potential for identifying active HDV infections. Importantly, serum samples with viral target Cts ≤ 30, indicative of moderate-to-high HDV RNA levels, were selected for evaluation. While this approach optimised assay performance, it may limit generalisability, as the ability to detect infections with lower viral loads remains a critical aspect of early diagnosis and disease management. Addressing this limitation in future studies will be essential to assess the assay's sensitivity across a broader range of viral loads, ensuring its applicability to diverse clinical contexts.

Genotypic analysis of the tested samples revealed that all sequenced HDV isolates belonged to genotype 3, which is predominantly found in South America, particularly in the western Amazon basin. Genotype 3 is associated with the most severe liver disease^38^ manifestations and poses significant public health challenges in the region. The developed assay was specifically designed and validated using genotype 3 samples, achieving high sensitivity and specificity within this local context. While these findings highlight the assay's diagnostic potential in regions where genotype 3 is prevalent, its genotype-specific validation may limit applicability in other epidemiological settings. Variations among HDV genotypes could affect assay performance, emphasising the need for additional studies to evaluate its efficacy across diverse genotypes. Expanding validation efforts to include other genotypes is crucial for broadening the assay's utility and ensuring its relevance in various global scenarios.

Although the rHDV-based ELISA showed promising diagnostic performance under controlled conditions, further studies are warranted to assess its applicability in large-scale screening and routine surveillance settings. Since the assay validation was performed using serum samples obtained from a public diagnostic laboratory in the Brazilian Amazon region and derived from infections associated with a single HDV genotype (HDV-3), which predominates in this region, it is important to conduct future studies to evaluate the assay's performance against other HDV genotypes and to assess the extrapolation of results to other populations or epidemiological contexts. The inclusion of larger and more diverse cohorts from endemic regions will help to further support the robustness and broader applicability of this diagnostic approach.

This study demonstrated that the immunoenzymatic platform based on the HDV mimetic molecule, developed using *phage display* technology, represents a viable alternative for HDV detection. The recombinant rHDV protein achieved an AUC of 0.9665, with 88% sensitivity and 98.92% specificity, highlighting its potential for detecting active hepatitis delta infections, particularly in resource-constrained settings. Although the assay does not match the diagnostic accuracy of commercial kits, it offers a cost-effective and accessible solution that could facilitate early diagnosis and expand testing availability in underserved regions, such as the Amazon, where genotype 3 predominates and HDV burden is high. Despite its limitations, the assay's affordability and adaptability make it a valuable complement to existing diagnostic approaches, addressing gaps in healthcare access and supporting efforts to mitigate the impact of hepatitis delta in vulnerable populations.

## SUPPLEMENTARY MATERIALS

Supplementary data

## Data Availability

The HDV sequences are available in the GenBank repository, with accession numbers described in Supplementary data (Table II). All datasets generated and/or analysed during the current study are available from the corresponding author upon request.
