# Supplementary material for: Novel epitope-based diagnostic probes selected by phage display for the serological detection of HDV
Source: Mem Inst Oswaldo Cruz. 2026 Jun 12;121:e250343. doi: 10.1590/0074-02760250343 (PMC13262798; doi:10.1590/0074-02760250343)
Supplement: Supplementary data [file 1678-8060-mioc-121-e250343-s1.pdf]

TABLE I

Sequence and frequency of phage clones

Clones	Sequence	Frequency clones
HD1	KQEDGGP	1/80
HD2	KDAMGGP	5/80
HD3	RNEDGGP	3/80
HD4	QQEDGGP	18/80
HD5	NRDHRIQ	1/80
HD6	KDNTGGP	1/80
HD7	PRQEDGG	1/80
HD8	MNESFPG	1/80
HD9	KDEFGAY	3/80
HD10	SKQEDGG	2/80
HD11	SKEATPF	1/80
HD12	KDEHAGS	1/80
HD13	KEDQRGQ	1/80
HD14	TSEDGGP	1/80
HD15	KDGAGGP	2/80
HD16	DTTLHLG	2/80
HD17	KDSVSGP	1/80
HD18	KDNPGGP	1/80
HD19	KDAVGGP	4/80
HD20	KDSVAGP	1/80
HD21	YLHGYGT	1/80
HD22	LWWEKPT	1/80
HD23	LWWEKQK	1/80
HD24	LWWQTHL	1/80
HD25	RSDDGGP	2/80
HD26	KAYPYLK	1/80
HD27	RDSHRLL	1/80
HD28	DTTLSDN	1/80
HD29	KEDHVGP	1/80
HD30	QQEDGGH	1/80
HD31	KDDAGGP	3/80
HD32	KDEYGAY	1/80
HD33	NSHRHGA	1/80
HD34	DTTLTWF	1/80
HD35	KDESGSY	1/80
HD36	TQKEPAW	1/80
HD37	RYDDGGP	1/80
HD38	MSTDNNY	1/80
HD39	KDGSGGP	1/80
HD40	MEEDGGP	1/80
HD41	LTDVRGA	1/80
HD42	KEGHECQ	1/80
HD43	LWWEQSR	1/80
HD44	KDGLAGP	1/80
HD45	KDPGAGP	1/80
HD46	SRIQMLH	1/80

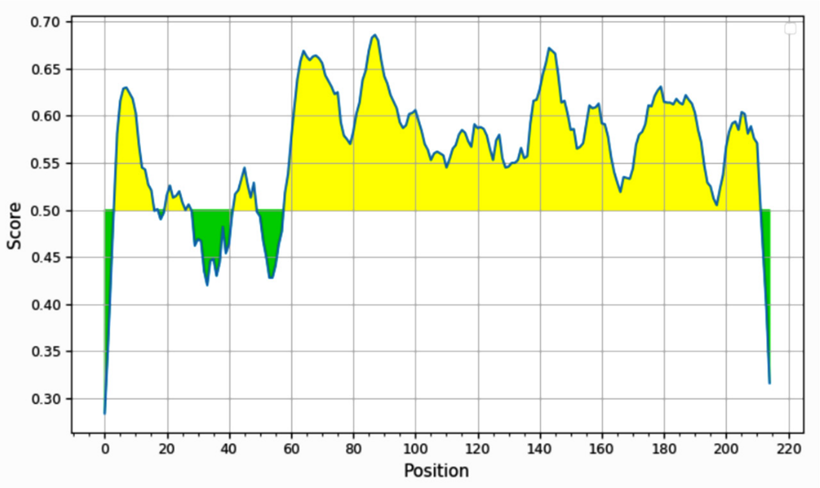

Fig. 1: antigenicity analysis of the large hepatitis delta antigen (L-HDAg) protein sequence using the Bepipred program. The yellow peaks represent B-cell epitopes, while the green peaks represent non-B-cell epitopes.

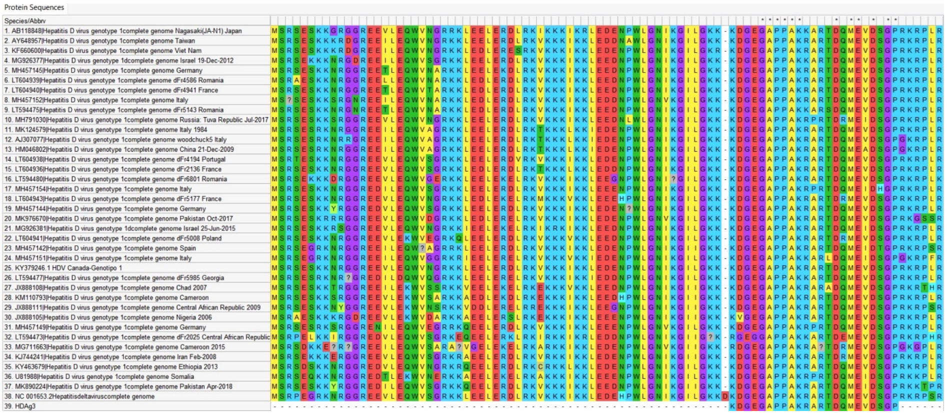

Fig. 2: alignment containing 38 hepatitis delta virus 1 (HDV-1) sequences from different locations and the hepatitis delta antigen 3 (HDAg3) epitope used in the study. The binding region corresponds to positions 63-85 aa of the large HDAg (L-HDAg) antigen in the reference sequence NC_001653.

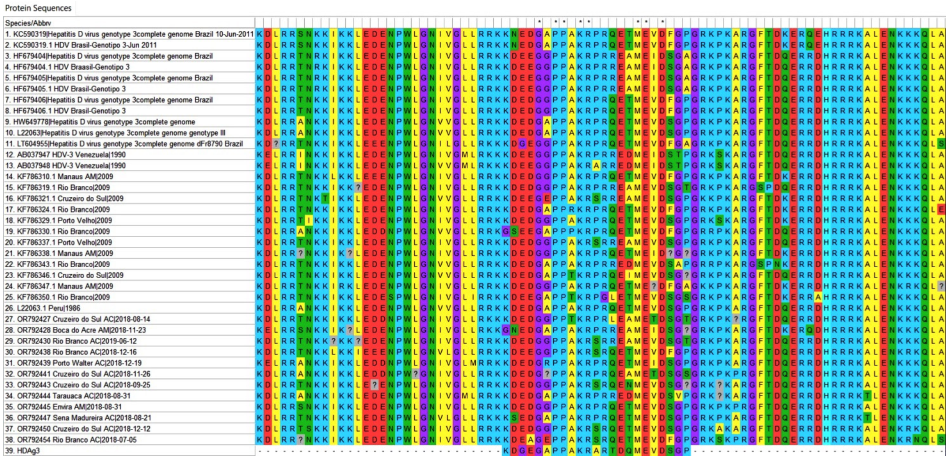

Fig. 3: alignment containing 38 hepatitis delta virus 3 (HDV-3) sequences from different locations and the hepatitis delta antigen 3 (HDAg3) epitope used in the study. The binding region corresponds to positions 63-85 aa of the large HDAg (L-HDAg) antigen in the reference sequence NC_001653.

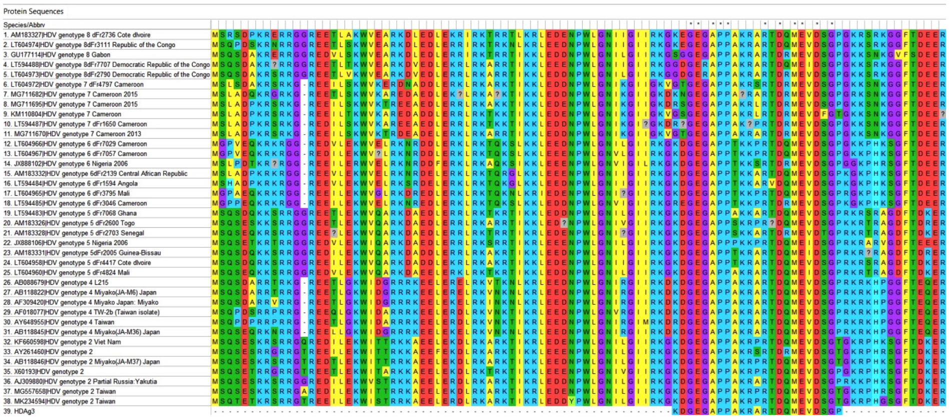

Fig. 4: alignment containing 38 hepatitis delta virus 2 (HDV-2), HDV-4, HDV-5, HDV-6, HDV-7, and HDV-8 sequences from different locations and the hepatitis delta antigen 3 (HDAg3) epitope used in the study. The binding region corresponds to positions 63-85 aa of the large HDAg (L-HDAg) antigen in the reference sequence NC_001653.

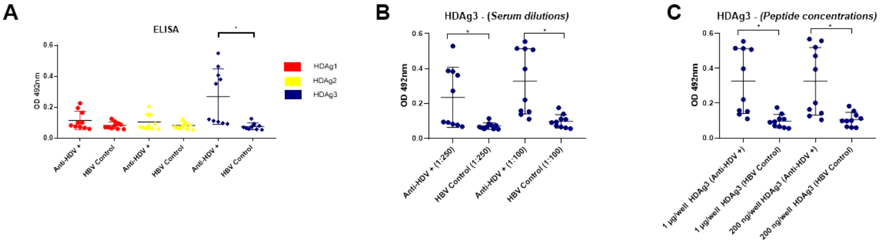

Fig. 5: enzyme-linked immunosorbent assay (ELISA) to evaluate synthetic peptides. (A) Analysis of different synthetic peptides: hepatitis delta antigen 1 (HDAg1), HDAg2, and HDAg3; (B) Assessment of different serum dilutions (1:100 and 1:250) with HDAg3; (C) Comparison of different HDAg3 concentrations (200 ng/well and 1 µg/well) in serum samples from 10 anti-hepatitis delta virus (HDV)+ individuals and 10 hepatitis B virus (HBV) control individuals.

TABLE II
Genotype of hepatitis delta virus (HDV) sample, epidemiological data, and viral load of HDV

ID sample	Viral load (Log 10 copies/mL)	Collection date	Residence city	*GenBank*	HDV genotype
4.3	6,02	14/08/2018	Cruzeiro do Sul -AC	OR792427	HDV-3
4.4	6,38	23/11/2018	Boca do Acre - AM	OR792428	HDV-3
4.9	3,69	29/10/2018	Guajará- AM	OR792429	HDV-3
4.18	4,69	12/06/2019	Rio Branco - AC	OR792430	HDV-3
4.20	6,50	22/02/2019	Rio Branco - AC	OR792431	HDV-3
4.23	5,36	28/01/2019	Rio Branco - AC	OR792432	HDV-3
4.27	6,52	19/12/2018	Rio Branco - AC	OR792433	HDV-3
4.31	5,27	11/10/2018	Rio Branco - AC	OR792434	HDV-3
4.38	5,04	26/07/2018	Rio Branco - AC	OR792435	HDV-3
4.39	7,72	11/07/2018	Rio Branco - AC	OR792436	HDV-3
4.48	5,05	13/04/2018	Rio Branco - AC	OR792437	HDV-3
4.52	6,37	16/12/2018	Rio Branco - AC	OR792438	HDV-3
4.54	6,40	19/12/2018	Porto Walter - AC	OR792439	HDV-3
4.56	4,46	08/01/2019	Cruzeiro do Sul - AC	OR792440	HDV-3
4.59	5,98	26/11/2018	Cruzeiro do Sul - AC	OR792441	HDV-3
4.71	5,99	31/10/2018	Cruzeiro do Sul - AC	OR792442	HDV-3
4.74	4,70	25/09/2018	Cruzeiro do Sul - AC	OR792443	HDV-3
4.76	7,68	31/08/2018	Tarauacá - AC	OR792444	HDV-3
4.77	6,10	31/08/2018	Envira - AM	OR792445	HDV-3
4.81	5,10	27/08/2018	Cruzeiro do Sul - AC	OR792446	HDV-3
4.82	6,17	21/08/2018	Sena Madureira - AC	OR792447	HDV-3
4.84	6,83	09/08/2018	Porto Walter - AC	OR792448	HDV-3
4.87	6,59	09/07/2018	Feijó - AC	OR792449	HDV-3
4.93	4,75	12/12/2018	Cruzeiro do Sul - AC	OR792450	HDV-3
4.94	6,33	03/08/2018	Sena Madureira - AC	OR792451	HDV-3
4.96	4,61	07/11/2018	Cruzeiro do Sul - AC	OR792452	HDV-3
4.98	5,93	06/11/2018	Porto Walter - AC	OR792453	HDV-3
4.105	5,62	05/07/2018	Rio Branco - AC	OR792454	HDV-3

AC: Acre; AM: Amazonas.
