# Supplementary material for: Novel epitope-based diagnostic probes selected by phage display for the serological detection of HDV
Source: Mem Inst Oswaldo Cruz. 2026 Jun 12;121:e250343. doi: 10.1590/0074-02760250343 (PMC13262798; doi:10.1590/0074-02760250343)
Supplement: Peer review history

# Reviewer report: FIRST REVIEW ROUND - REVIEWERS COMMENTS

## REVIEWER #1

I would like to thank you for the opportunity to review the manuscript. This is a topic of great relevance, given the proposal of a new immunoassay for detecting anti-HDV antibodies on a low-cost platform with good sensitivity and specificity parameters. The manuscript is well written but requires some adjustments before publication. Below are my comments:

**In the Abstract:** I suggest that the authors revise the research justification in the background (if there is room to increase the character count in this section), by mentioning underdiagnosis and the need for new serological methods for large-scale screening, particularly in endemic regions, rather than merely citing a “gap in testing for HDV among patients.”

**In the Introduction:**

a) In the third paragraph, the authors could emphasize the prevalence of infection in the Amazon region of Brazil immediately after reference 7 (line 31, page 5).

b) Review the spelling of the term “Phage display,” as it appears in regular text at times (line 38) and in italics at others (line 46, page 5).

**In Methods:**

a) Revise the phrasing “A total of 200 residual blood serum samples” and consider whether “A total of 200 blood serum samples” might be more appropriate.

**In Results:**

a) In section 3.4, the authors mention two serum dilutions and two concentrations of HDAg3 but do not specify which yielded better results.

b) In Figure 5, there are brackets with asterisks, but no explanation is provided in the caption. The authors should include a corresponding note.

**In the Discussion:**

a) The authors should add a paragraph outlining the limitations of the study.

## AUTHORS’ RESPONSE TO THE REVIEWERS

**To reviewer #1:**

I would like to thank you for the opportunity to review the manuscript. This is a topic of great relevance, given the proposal of a new immunoassay for detecting anti-HDV antibodies on a low-cost platform with good sensitivity and specificity parameters. The manuscript is well written but requires some adjustments before publication. Below are my comments:

**In the Abstract:** I suggest that the authors revise the research justification in the background (if there is room to increase the character count in this section), by mentioning underdiagnosis and the need for new serological methods for large-scale screening, particularly in endemic regions, rather than merely citing a “gap in testing for HDV among patients.”

**Response:** Thank you for your insightful comment. We have revised the abstract to incorporate your suggestion, while maintaining the journal’s required word limit. The new version of the abstract is presented below; The same changes has been added to the last version of this manuscript.

BACKGROUND: HDV is associated with the worst prognosis among viral hepatitis infections however; it remains largely underdiagnosed, particularly in developing endemic regions, underscoring the need for new, accessible, and appropriate serological methods for large-scale screening.

OBJECTIVES: To develop HDV-mimetic molecules using phage display for application in immunodiagnostic platforms.

METHODS: HDV-mimetic peptides were selected via phage display biopanning, sequenced, and screened by phage-ELISA. Based on the sequences of these peptides, a recombinant protein (rHDV) was constructed and utilized in an Enzyme-Linked Immunosorbent Assay (ELISA). The test was validated using 87 anti-HDV-positive samples and 93 HBV control samples collected from a public diagnostic laboratory in the Amazon region. Statistical analyses were performed to evaluate the diagnostic performance of the synthetic peptides and the recombinant protein.

FINDINGS: Overall, rHDV exhibits a sensitivity of 74.71%, specificity of 97.85%, and AUC of 0.8906. In HDV RNA-positive patients, diagnostic performance improved, with a sensitivity of 88.0%, specificity of 98.92%, and AUC of 0.96.

MAIN CONCLUSIONS: These findings highlight that the rHDV protein used in ELISA effectively discriminated HDV-infected individuals from patients monoinfected with hepatitis B. This demonstrates the potential of rHDV as an effective, fast, and low-cost tool for HDV detection, for broader HDV screening.

**-In the Introduction:**

a) In the third paragraph, the authors could emphasize the prevalence of infection in the Amazon region of Brazil immediately after reference 7 (line 31, page 5).

**Response:** We appreciate the suggestion, the third paragraph was revised to emphasize the high prevalence of HDV in the Brazilian Amazon region following reference 7, as suggested.

Chronic hepatitis D infection is the most severe form of viral hepatitis, with a high risk of progression to liver cirrhosis, hepatocellular carcinoma, and liver-related death5,6. Current guidelines recommend HDV testing for all patients with chronic HBV infection7. However, there is a considerable lack of testing, particularly in developing countries where the virus is endemic8, leading to its underestimation7. Notably, high HDV prevalence has been reported in the Brazilian Amazon region 9–13, with approximately 72.4% of all the cases reported between 2000 and 2024 occurring in the Northern region of Brazil, which is also the source of the samples analyzed in this study14. These challenges highlight the urgent need for affordable and accessible tools for HDV screening.

b) Review the spelling of the term “Phage display,” as it appears in regular text at times (line 38) and in italics at others (line 46, page 5).

**Response:** We appreciate your recommendations. The term “*phage display*” was updated and italicized in the last version of this manuscript.

**-In Methods:**

a) Revise the phrasing “A total of 200 residual blood serum samples” and consider whether “A total of 200 blood serum samples” might be more appropriate.

**Response:** Thank you for your suggestion. We will revise the manuscript accordingly.

**-In Results:**

a) In section 3.4, the authors mention two serum dilutions and two concentrations of HDAg3 but do not specify which yielded better results.

**Response:** We appreciate the reviewer’s observation. The paragraph has been revised to clarify which serum dilution and HDAg3 concentration yielded the most suitable results, as detailed below.

The reactivity of the three synthetic peptides (HDAg1, HDAg2, and HDAg3) was compared via ELISA, and we observed that the HDAg3 peptide exhibited superior reactivity. Therefore, the HDAg3 peptide was selected for use in subsequent experiments. Two different serum dilutions (1:100 and 1:250) and two HDAg3 concentrations (200 ng/well and 1 μg/well) were evaluated using 10 anti-HDV-positive serum samples and 10 HBV control samples for test standardization (Supplementary Fig. 5). Both serum dilutions and antigen concentrations showed comparable and satisfactory performance in differentiating HBV from anti-HDV controls. Given the equivalent performance, the lower antigen concentration (200 ng/well) and the higher serum dilution (1:250) were selected to minimize reagent and human serum consumption. As a result, the synthetic peptide HDAg3 successfully differentiated pooled serum samples from anti-HDV-positive patients and hepatitis B patients.

b) In Figure 5, there are brackets with asterisks, but no explanation is provided in the caption. The authors should include a corresponding note.

**Response:** Thank you for the reviewer’s comment. Brackets with asterisks were used to denote statistically significant differences between the HBV control group and the anti-HDV–positive group. The following note will be added to the figure legend:

Note: (*) Statistically significant difference.

**-In the Discussion:**

a)The authors should add a paragraph outlining the limitations of the study.

**Response:** We thank you again and agree to hightlight the limitations of this study. It’s been added this paragraph to the new version of the manuscript.

Although the rHDV-based ELISA showed promising diagnostic performance under controlled conditions, further studies are warranted to assess its applicability in large-scale screening and routine surveillance settings. Since the assay validation was performed using serum samples obtained from a public diagnostic laboratory in the Brazilian Amazon region and derived from infections associated with a single HDV genotype (HDV-3), which predominates in this region, it is important to conduct future studies to evaluate the assay’s performance against other HDV genotypes and to assess the extrapolation of results to other populations or epidemiological contexts. The inclusion of larger and more diverse cohorts from endemic regions will help to further support the robustness and broader applicability of this diagnostic approach.

# Reviewer report: REVIEWERS COMMENTS

## REVIEWER #1

All suggested modifications have been incorporated into the final version; therefore, I recommend the manuscript for publication.
